# Tailoring conductive inverse opal films with anisotropic elliptical porous patterns for nerve cell orientation

**DOI:** 10.1186/s12951-022-01340-w

**Published:** 2022-03-09

**Authors:** Zeyou Zhang, Yu Wang, Zhuoyue Chen, Dongyu Xu, Dagan Zhang, Fengyuan Wang, Yuanjin Zhao

**Affiliations:** 1 Department of Clinical Laboratory, Institute of Translational Medicine, The Affiliated Drum Tower Hospital of Nanjing University Medical School, Nanjing, 210008 China; 2 State Key Laboratory of Bioelectronics, School of Biological Science and Medical Engineering, Southeast University, Nanjing, 210096 China; 3 Department of Dermatology, Zhongda Hospital, Southeast University, Nanjing, 210009 China

**Keywords:** Inverse opal, Conductive, Anisotropic, Nerve orientation, Hydrogel

## Abstract

**Background:**

The nervous system is critical to the operation of various organs and systems, while novel methods with designable neural induction remain to exploit.

**Results:**

Here, we present a conductive inverse opal film with anisotropic elliptical porous patterns for nerve orientation induction. The films are fabricated based on polystyrene (PS) inverse opal scaffolds with periodical elliptical porous structure and poly(3,4-ethylenedioxythiophene):poly(styrenesulfonate) (PEDOT:PSS) mixed polyacrylamide (PAAm) polymers fillers. It is demonstrated that the anisotropic elliptical surface topography allows the nerve cells to be induced into orientation connected with the stretching direction. Because of the anisotropic features of the film which can be stretched into different directions, nerve cells can be induced to grow in one or two directions, forming a neural network and promoting the connection of nerve cells. It is worth mentioning that the PEDOT:PSS-doped PAAm hydrogels endow the film with conductive properties, which makes the composite films be a suitable candidate for neurites growth and differentiation.

**Conclusions:**

All these features of the conductive and anisotropic inverse opal films imply their great prospects in biomedical applications.

**Graphical Abstract:**

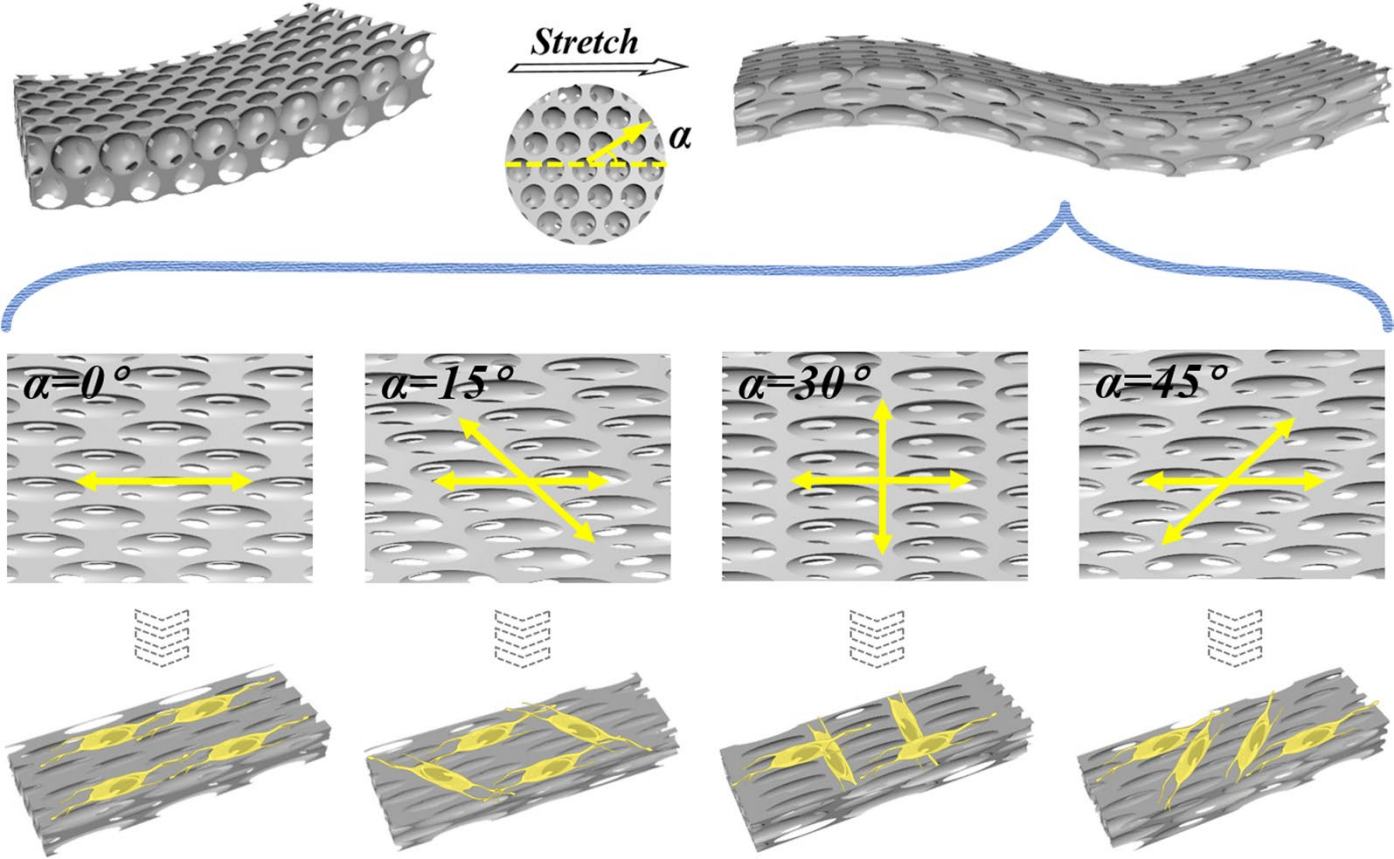

**Supplementary Information:**

The online version contains supplementary material available at 10.1186/s12951-022-01340-w.

## Background

The nervous system, a potentially central role in human life, can appropriately regulate the body’s physiological functions towards environmental changes to achieve harmony between organism and environment [1–3]. Thus, the neuronic injury and retrogression can lead to permanent tissue injury and severe functional damage, which is unavoidable to human beings [4, 5]. However, the inherent self-regeneration ability of nerve tissues is limited, so that the repairing and regeneration of damaged nerve tissues have attracted remarkable attention [6–8]. Numerous studies have been devoted to solving these issues, in which nerve orientation induction is regarded as an effective way [9–16]. In this aspect, the substrate materials with surface morphology have been demonstrated with great potential in nerve orientation [17, 18]. Despite many progresses have been achieved by employing the surface morphology biomaterials [19–22], there remain challenges in achieving neuron induction due to the softness of these biomaterials [23]. In contrast, stiff patterned substrates exhibit induced-orientation potentials [8], while the inconsistency between substrates and the in vivo organism usually hinders the survival and proliferation of nerve cells [24]. Additionally, most of the recently single orientation induction is not conducive to the rapid formation of neural network [19]. Thus, new methods with designable neural induction are still anticipated for constructing complex neural system.

**Fig. 1:**
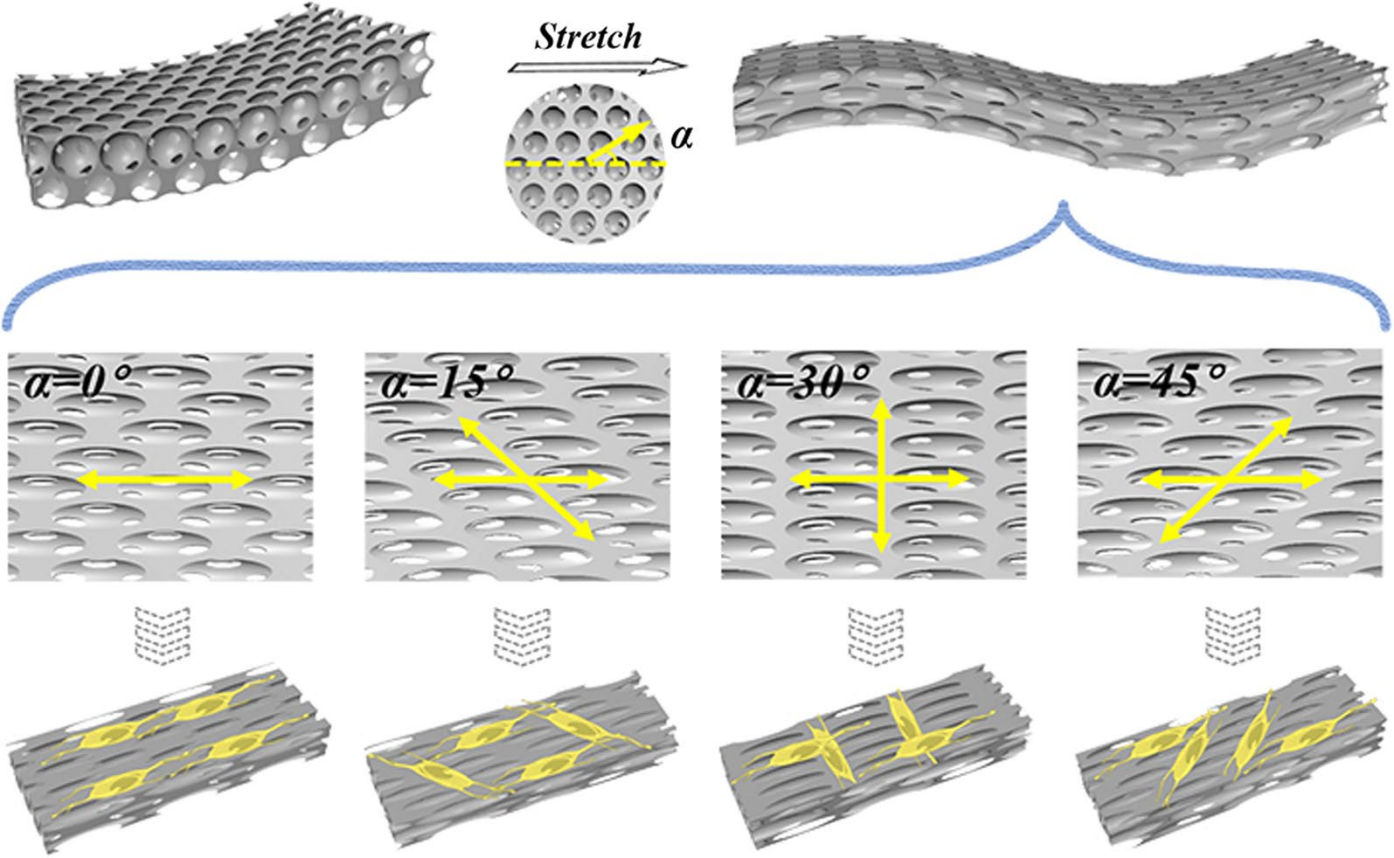
Scheme of tailored inverse opal films with different stretching directions for nerve cells induction. “*α*” means the angle between the stretching direction and the straight line of the single row of holes. Nerve cells were allowed to be induced into orientations connected with the surface topography, forming neural networks with different crossing degrees

In this paper, we proposed a novel conductive and anisotropic inverse opal substrate with anisotropic elliptical porous patterns for nerve orientation induction, as schemed in Fig. 1. Inverse opals, originated from colloidal crystal array templates, are structured materials with three-dimensional (3D), periodically arranged nano/micro-pores [25–27]. It uniquely features in the long-range ordered structure, uniform and well-controlled pore sizes, and homogeneous interconnectivity [27–29]. These characteristics allow the inverse opals to be explored in numerous biomedical applications [30–33]. Attractively, when the inverse opals were stretched, the stretched substrate with anisotropic elliptical porous patterns gained potentials to affect cell adhesion and proliferation, on which the cells would grow in orientation related to the stretching direction [34, 35]. Specially, the surface morphology of the inverse opals will change with the tensile angle, which is manifested in the different arrangement of elliptical holes [36, 37]. In addition, the inverse opals could be further potentially integrated with conductive materials, which benefit the regulation of neuronal proliferation and differentiation, as well as nerve tissue regeneration and reconstruction [38–44]. However, the conductive materials integrated inverse opal structures and their applications in controllable nerve cell induction and neural network construction are rarely reported yet.

Herein, we tailored a conductive inverse opal film with anisotropic elliptical porous patterns for inducing nerve cells in a specific direction and interaction. The films were composed of polystyrene (PS) inverse opal scaffolds and poly(3,4-ethylenedioxythiophene):poly(styrenesulfonate) (PEDOT:PSS) mixed polyacrylamide (PAAm) polymers fillers. As the PS film could be stretched to a specific proportion of its original length in different directions, the derived anisotropic properties of the film enabled the nerve cells to grow in a certain direction connected with the stretching direction. Because the nerve cells would grow along one or two directions formed by the stretched substrates, it was quite convenient to induce nerve cells to grow in different degrees of interlace simply by changing the stretching direction. In addition, the introduction of PEDOT:PSS-doped PAAm hydrogels provided conductivity, making the composite film more suitable for neurites to grow and extend in their differentiation. These features indicated that the proposed stretched inverse opal and conductive hydrogels composite film had broad prospects in biomedical applications, especially in neural tissue engineering.

## Results and discussion

**Fig. 2:**
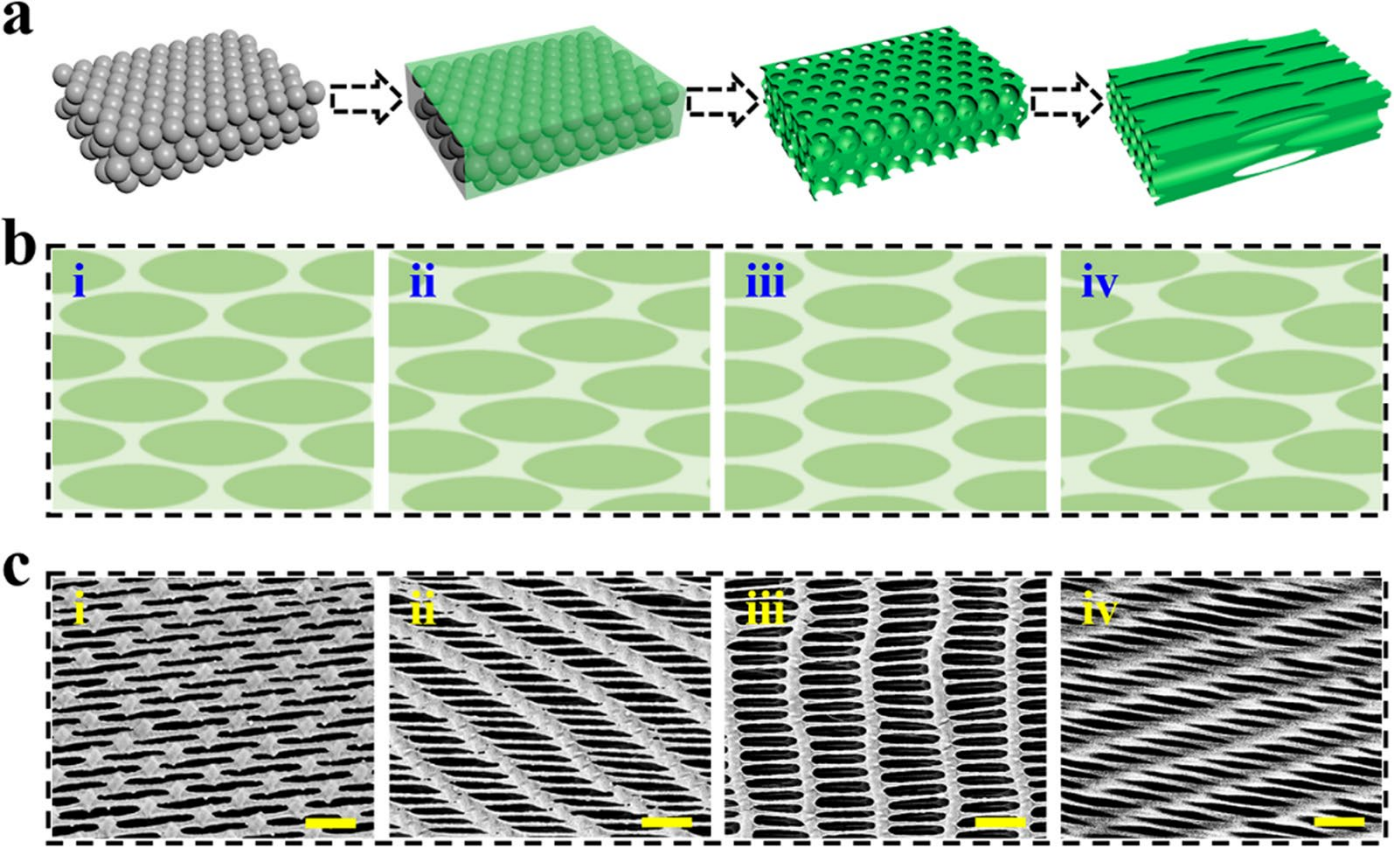
Preparation process and characterization of surface morphology of the inverse opal substrates stretched at different directions. **a** Schemes of the fabrication procedures of the stretched inverse opal scaffolds (take the stretching angle of 0° as an example). **b** Schematic diagrams and **c** SEM images of the scaffolds stretched at different angles to the straight line of the single row of holes: (i) 0°, (ii) 15°, (iii) 30°, (iv) 45°. All these substrates were stretched 6 times of their initial lengths. Scale bars are 500 nm in (**c**)

In a typical experiment, the anisotropic inverse opal substrates were originated from inverse opal scaffolds, which could be acquired by colloidal crystal templates through a vertical deposition method (Fig. 2a). In detail, silica nanoparticles could be self-assembled on the glass slides, which were perpendicular into an ethanol solution of monodisperse silica nanoparticles. With the gradual volatilization of ethanol solvent, silica templates with hexagonal close-packed structures were formed (Additional file 1: Fig. S1a). The as-prepared templates were sintered at 500 °C to enhance the mechanical strength of the templates as well as the junction structures of SiO_2_ nanoparticles. Compared with other scaffold materials, PS possesses good biocompatibility as well as excellent mechanical properties, such as high hardness and outstanding rigidity. These characteristics enable PS to support the growth of cells after being stretched, therefore, it was selected as a suitable scaffold material to construct inverse opal structure films. First, a pregel PS / toluene solution was injected into the holes of the SiO_2_ templates. After the evaporation of toluene at room temperature, a composite structured substrate embedding with silica nanoparticles was acquired (Additional file 1: Fig. S1b). Finally, the PS substrates with inverse opal structures were obtained by etching SiO_2_ colloidal crystal arrays with hydrofluoric acid (HF) (Additional file 1: Fig. S1c).

To endow the PS inverse opal scaffolds with anisotropic features, the substrates were stretched at different angles to the straight line of the single row of holes. Generally, stretching degrees had an obvious impact on the morphologies and properties of PS inverse opal scaffolds substrates. Thus, inverse opal substrates with different stretching degrees were first investigated. It could be found that the PS inverse opal nanopores changed from circle to ellipse with the increase of tensile strength and with the increase of stretching degrees, the deformation of the nanoscale pores was larger (Additional file 1: Fig. S2). It has been demonstrated that the greater the degree of stretching, the better the induction performance of the substrates [35]. However, when the stretching degree exceeded 6 times, the elliptical pore structures of the inverse opal film would be destroyed, thereby destroying its long-range ordered structure and damaging the induction of cells (Additional file 1: Fig. S2c, d). Therefore, stretching degree of 6 times was used for the following experiments. Because of hexagonal close-packed structures of silica templates, the PS scaffolds could be stretched at four angle directions, including 0°, 15°, 30° and 45° to the straight line of the single row of holes and four kinds of inverse opal substrates with anisotropic features would be obtained (Fig. 2b). The surface morphologies of four kinds of inverse opal substrates were studied (Fig. 2c). When stretched at 0°, the periodic ellipse-nanopores of scaffolds arranged along the stretching direction (Fig. 2ci).

In the condition of 15°, 30° and 45° stretching directions, similar phenomena were observed; in comparison, evident ridges appeared, the difference of which was the angles between the ridges and the stretching directions (Fig. 2cii–iv). The angles between the trend caused by ridges and the stretching direction on 15°, 30° and 45°-stretched substrates were acute angle, right angle and acute angle, respectively. In the case of 15°, 30° and 45° stretching directions, two orientations occurred. In addition to the orientation along with the stretching direction, another orientation was related to the emergence of evident ridges. The difference between them was that the angles between the two directions were acute, right, and acute, respectively. Besides, the orientations of ridges on 15° and 45°-stretched substrates were facing opposite directions. The prepared stretched inverse opal substrates with unique anisotropic characteristics provided a new direction for cell directional growth and arrangement.

To investigate the potential of the stretched scaffolds in inducing nerve cells, four kinds of inverse opal substrates with anisotropic features were adopted as the culture substrates for the neuronal cell, PC12 cells, a commonly used nerve cell strain. They will differentiate into cells with sympathetic neuron characteristics under the induction of nerve growth factor (NGF), which has an essential effect on the study of neuronal differentiation and mechanism of action [18]. First of all, the biocompatibility of the prepared substrates was tested. Ordinary glass slides were utilized as the blank control group. The activity of PC12 cells on films with different stretching angles was quantitatively investigated by 3-(4,5-dimethyl-2-thiazolyl)-2,5-diphenyltetrazolium bromide (MTT) analysis, as shown in Additional file 1: Fig. S3a. It was evident that all of these films demonstrated good biocompatibility. Additionally, the difference of substrates stretching angles had no effect on biocompatibility. Meanwhile, the anisotropic inverse opal substrates exhibited excellent cell adhesion (Additional file 1: Fig. S3b). These results indicated that the prepared substrates with distinct stretching angles would be suitable for cells growth.

**Fig. 3:**
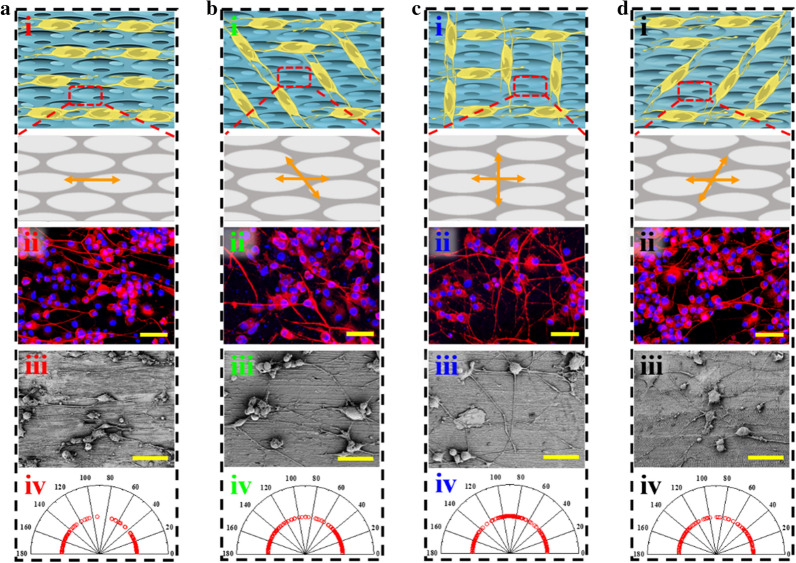
Cell culture on inverse opal films stretched at different directions. **a** PC12 cells cultured on 0°-stretched inverse opal films: (i) Schematic diagrams, (ii) immunofluorescence image, (iii) SEM image, and (iv) angle distribution of neurites of PC12 cells. **b** PC12 cells cultured on 15°-stretched inverse opal films: (i) Schematic diagrams, (ii) immunofluorescence image, (iii) SEM image, and (iv) angle distribution of neurites of PC12 cells. **c** PC12 cells cultured on 30°-stretched inverse opal films: (i) Schematic diagrams, (ii) immunofluorescence image, (iii) SEM image, and (iv) angle distribution of neurites of PC12 cells. **d** PC12 cells cultured on 45°-stretched inverse opal films: (i) Schematic diagrams, (ii) immunofluorescence image, (iii) SEM image, and (iv) angle distribution of neurites of PC12 cells. Scale bars are 50 μm in all (ii) and (iii)

Given these excellent features, inverse opal scaffolds with different stretching angles were cultured with PC12 cells and the effect of surface topography on the growth of PC12 cells was further studied (Fig. 3). To clearly observe PC12 cells behavior, neurons were stained red by a mouse anti-β III-tubulin primary antibody, and the nuclei were highlighted by 4’, 6-diamidino-2-phenylindole (DAPI) as blue. Compared with cells grown on ordinary glass slides which showed disordered microfilaments (Additional file 1: Fig. S4a, b), the neurons were arranged in specific directions on those stretched films. Precisely, when the films were stretched at 0°, the cells tended to grow along the stretching direction (Fig. 3aii), while the extension of the neurons had two growth orientations on films stretched at 15°, 30° and 45° (Fig. 3bii, cii, dii). Besides one along the stretching direction, another orientation was almost consistent with the direction of the ridges as excepted. SEM images demonstrated more clearly about the relationship between the surface morphology and the growing direction of neurons, which formed neural networks profiting from the surface morphology of the prepared PS films with different stretching directions (Fig. 3aiii, biii, 3ciii, diii). These features revealed that the stretched inverse opal scaffolds with elliptical topography could effectively induce PC12 cells into specific orientations.

To further investigate the cell orientation in response to four kinds of inverse opal substrates, the angles between the path of neurons and the stretching direction of substrates were detected and counted by ImageJ software package (Fig. 3aiv, biv, civ, div). The range of orientation angle is 0° ~ 180°. 0° and 180° indicate that the neurons' directions are the same as the stretching direction of the films, while 90° means the direction of neurons is perpendicular to the stretching direction (Additional file 1: Fig. S5). Compared with cells on ordinary glass slides which showed angle variations in all directions (Additional file 1: Fig. S4c), cells on four kinds of substrates all presented characteristic angle changes. Specifically, when it came to the 0°-stretched films, a majority of cells showed a noticeable orientation around 0° and 180° (Fig. 3aiv). Similarly, there was no accident that cells gathered below 40° and above 140° on films stretched at 15° and 45° and around 0°, 90° and 180° on 30°-stretched films (Fig. 3biv, civ, div). The corresponding angle analysis was conducted and investigated (Additional file 1: Fig. S5). It could be found that more than half of cells arranged around 0° and 180° on 0°-stretched films, while with regard to 15° and 45°-stretched films, about 75% of cells gathered below 40° and above 140°. Notably, the two arrangement directions were most distinct on films stretched at 30°, on which up to 35% and 30% of cells extended along and perpendicular to the stretching direction respectively. This might be ascribed to the small angle between the two directions of films stretched at 15° and 45° and the existence of measurement error. Therefore, 30°-stretched films were most suitable for cultivating a crossed neural network.

**Fig. 4:**
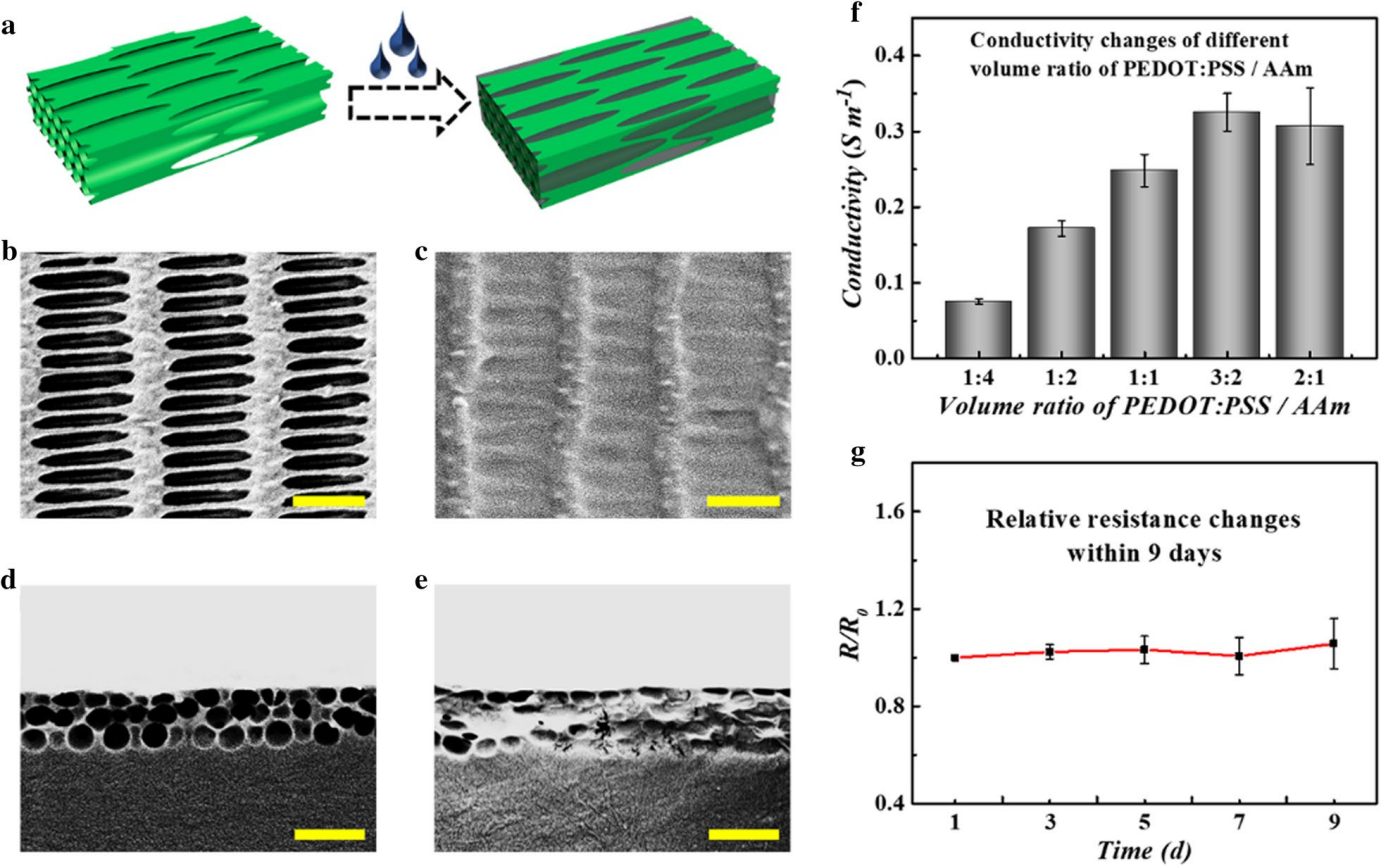
Preparation of the composite hydrogel film and characterization of its conductive properties. **a** Schematic diagrams of the perfusion process of PEDOT:PSS-doped PAAm hydrogels. **b** The SEM photograph of PS inverse opal film. **c** The SEM photograph of composite hydrogel film. **d** The SEM photograph of the cross-section of PS inverse opal film. **e** The SEM photograph of the cross-section of composite hydrogel film. **f** Conductivity changes of different volume ratios of PEDOT:PSS / AAm. **g** Relative resistance changes within 9 days. Scale bars are 1 μm in (**b**)-(**e**)

Biomaterials with great conductivity have been proven to exhibit a positive effect on neurite growth and differentiation due to the electrogenic properties of nerve cells, thus it would be an effective way for substrates to combine with conductive materials [45, 46]. Benefiting from its excellent properties including high conductivity, water stability and good biocompatibility [47], PEDOT:PSS was selected as conductive materials to impart inverse opal substrates with conductivity. In general, a pregel solution composed of PEDOT:PSS and AAm was prepared and injected into the micropores of the inverse opal substrates. After UV polymerization, a composite hydrogel film with an inverse opal layer and a conductive layer was obtained (Fig. 4a). The concentration of conductive layer was first optimized and analyzed. It could be seen that the conductivity of the films ascended as the concentration of PEDOT:PSS increased; while once the volume ratio of PEDOT:PSS / AAm reached 3:2, the opposite trend appeared (Fig. 4f). The trend appeared mainly ascribed to the fact that a high concentration of PEDOT:PSS can lead to agglomeration, which in turn caused a decrease in the conductivity of the films. Thus, the volume ratio of 3:2 was used for the construction of the composite hydrogel films, whose conductivity could reach 0.33 S m^−1^. In the process, the morphologies of the materials including before and after conductive layer infiltrated were observed (Fig. 4b–e). As expected, the conductive layer had completely filled the inverse opal voids, while without affecting their surface morphology. The results were consistent with Fig. 4c that the surface of the composite substrate was covered with conductive hydrogels, while the regularly arranged elliptical holes could still be seen. Besides, Raman spectrometer was further verified the existence of PEDOT:PSS in the composite hydrogel film, in which the peaks at 1433, 1368, 1258, 1100, 985, 854, 707, 574 and 444 cm^−1^ all belonged to PEDOT:PSS (Additional file 1: Fig. S6). Moreover, the conductivity stability of the composite hydrogel film was required to be investigated, which was vital due to the long culture time of cells. It was demonstrated that the resistance remained stable within up to 9 days, indicating the conductivity variation of the films could be negligible during cell culture (Fig. 4g).

**Fig. 5:**
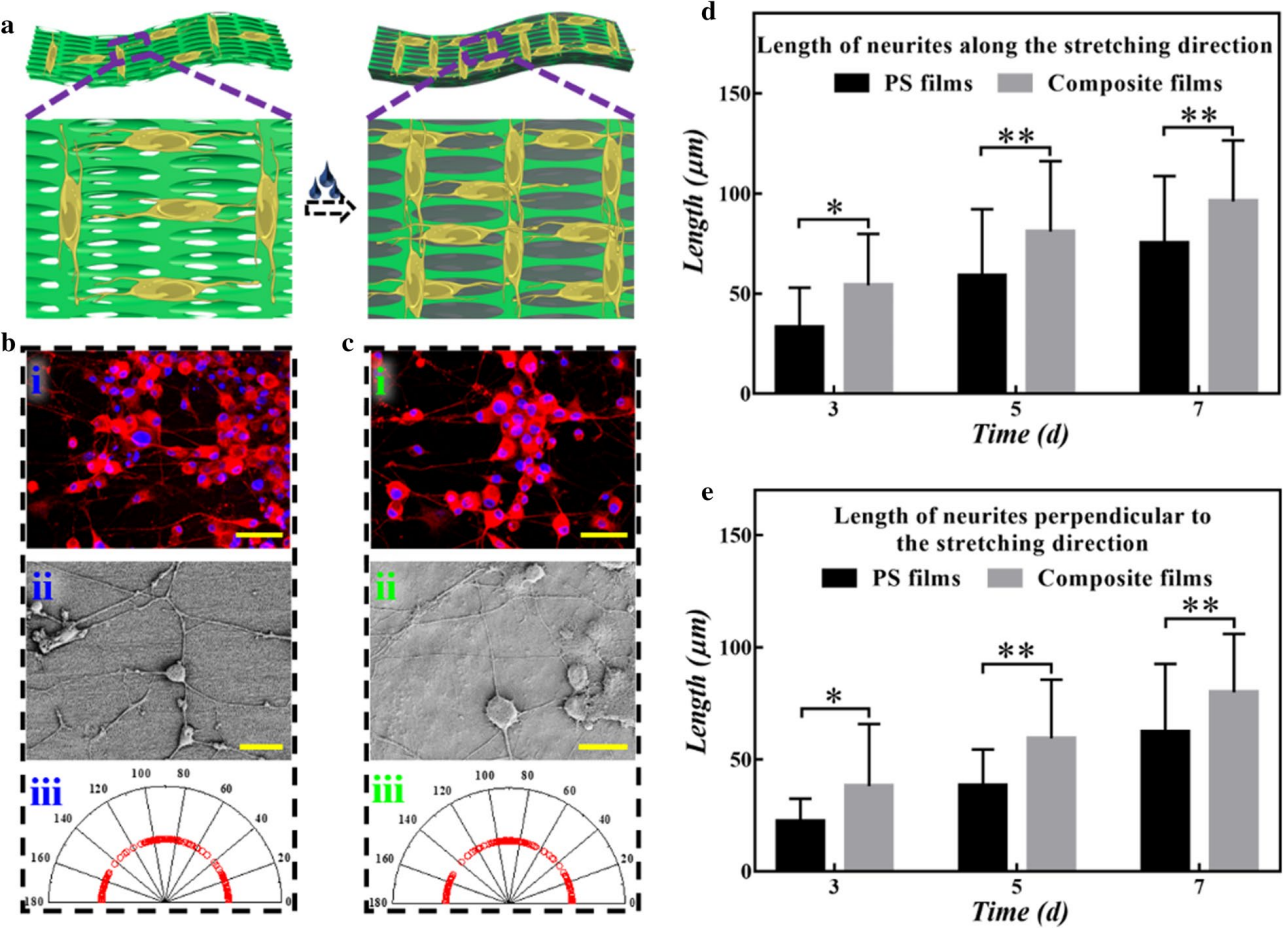
Cell culture on composite hydrogel films. **a** Schematic diagrams of PC12 cells cultured on PS inverse opal films and composite hydrogel films. **b** PC12 cells cultured on PS inverse opal films: (i) immunofluorescence image, (ii) SEM image, (iii) angle distribution of PC12 cells. **c** PC12 cells cultured on composite hydrogel films: (i) immunofluorescence image, (ii) SEM image, (iii) angle distribution of PC12 cells. **d** Length of neurites along the stretching direction changes with culture days. **e** Length of neurites perpendicular to the stretching direction changes with culture days. All these substrates were stretched at 30°. Scale bars are 50 μm in all (i) and (ii)

To evaluate the effect of conductivity on cell behavior, the composite hydrogel films were utilized as substrates for culturing PC12 cells (Fig. 5a). It could be found that the composite hydrogel films showed good biocompatibility and cell adhesion ability (Additional file 1: Fig. S7). Afterwards, PC12 cells were inoculated into these materials and cultured in the medium with NGF for 7 days. The concentration we used can most benefit the differentiation of PC12 cells. The differentiation rates of PC12 cells were first counted. As shown in Additional file 1: Fig. S8a, when it reached the 7th day, the differentiation rate of PC12 cells cultured on the composite hydrogel films displayed well due to the conductive hydrogel as well as the optimal concentration of NGF. Apparently, compared with which on the bare inverse opal substrates, the neurons on the composite hydrogel films showed better distribution and possessed longer neurites, exhibiting tightly connections with films according to immunofluorescence images (Fig. 5bi, ci). The SEM images (Fig. 5bii, cii) vividly revealed the relationship between the type of substrates and the growth of PC12 cells. It could be seen that taking the advantage of the conductive layer, the perpendicular neural network was formed and remained intact. Additionally, the network had a more sufficient interlock on composite films which had potential benefits to the connection and conduction of nerve signals. The direction angles of cells were also quantitatively and the results of angle distribution and statistics were consistent with the above results (Fig. 5biii, ciii). And the orientation distributions of cells on the composite hydrogel films and bare inverse opal substrates were similar (Additional file 1: Fig. S8b) which mean the cover of conductive hydrogel films exhibited negligible influence on the alignment of cells. Then, the length of neurites along and perpendicular to the stretching direction was measured to investigate the influence of the composite hydrogel films on the growth of PC12 cells (Fig. 5d, e). It could be seen that PC12 cells retained longer neurites on the composite hydrogel films compared to PS inverse opal substrates on both two directions over time. The average length of neurites along the stretching direction reached up to 96 μm while the neurites perpendicular to the stretching direction attained 80 μm on the composite hydrogel films compared with 75 μm and 62 μm respectively on the bare inverse opal films, which showed that the conductive hydrogel films promoted the growth of PC12 cells. These results demonstrated that the composite hydrogel films showed great advantages in the growth and differentiation of cells, indicating their potential as a substrate for tissue engineering, especially neural engineering.

## Methods

### Materials

Silica nanoparticles of diameters about 370 nm were synthesized in our laboratory. HF, toluene solution and ethanol were obtained from Aladdin (Shanghai, China). Dimethyl sulfoxide (DMSO), MTT, glutaraldehyde, Triton-X100, laminin, acrylamide (AAm), N,N’-methylenebisacrylamide (MBA) and 2-hydroxy-2-methyl-1-phenyl-1-propanone (HMPP) were acquired from Sigma (USA). The PEDOT:PSS suspension (Clevios PH1000) was bought from Heraeus Electronic Materials GmbH (Leverkusen, Germany). Phosphate buffered saline (PBS), fetal bovine serum (FBS), horse serum (HS), NGF, DAPI, RPMI1640 and penicillin–streptomycin were obtained from Gibco (Rockville, USA). Donkey anti-mouse IgG (H + L) Alexa Fluor 555 secondary antibodies were obtained from Invitrogen (Carlsbad, USA). Mouse anti-βIII-tubulin primary antibodies were achieved from Abcam (UK). DAKO fluorescence mounting medium was purchased from DAKO (Denmark). PC12 cell line was from Chinese Academy of Sciences. A Millipore Milli-Q system (Millipore, Bedford, MA) was used to provide deionized water which owns a resistivity higher than 18 MΩ·cm.

### Preparation of stretched PS inverse opal substrates

The colloidal crystal templates were first fabricated through a vertical deposition method. An ethanol solution of SiO_2_ nanoparticles (1.5 wt%) was prepared and poured into a deposition bottle. A glass slide was put into the bottle at 45 °C and invariant humidity for 4 days, thereby, silica templates were obtained, which were calcined at 500 °C for 5 h. Next, a 20 wt% PS / toluene solution was prepared and injected into the templates. After gradual removing the solvent by room temperature, the solution was polymerized. PS inverse opal scaffolds were obtained through the sacrificial method, which were achieved by etching templates with 4 wt% HF solution. The obtained PS inverse opal substrates with anisotropic features were tailored at 0°, 15°, 30° and 45° to the straight line of the single row of holes. By stretching the substrates under the above-mentioned angles in a 70 °C water bath, four kinds of stretched PS inverse opal substrates could be achieved. Besides, the deformation of PS inverse opal substrates could be adjusted at different stretching degrees, and the stretched PS inverse opal substrates at 3, 6, 9, 12 times of original films were acquired.

### Preparation of conductive PS inverse opal substrates

PAAm prepolymer solution was made by mixing AAm (0.2 g mL^−1^), MBA (3.45 wt% relative to AAm) and deionized water. Then, the prepolymer solution of PAAm was mixed with a certain concentration of PEDOT:PSS aqueous suspension as conductive fillers. The volume ratio of PEDOT:PSS and PAAm prepolymer solution was adjusted including 1:4, 1:2, 1:1, 3:2 and 2:1, respectively. Finally, after adding 1% (v/v) HMPP (photoinitiator), the mixture was dropped onto the stretched films and polymerized by an ultraviolet (UV) light-emitting diode (LED) curing system (OmniCure S1000) for 60 s, 90 s, 120 s, 150 s, and 180 s respectively to generate the conductive inverse opal substrates.

### Cell culture

Firstly, the prepared substrates and ordinary glasses were soaked in 75% ethanol solution for 6 h and irradiated with UV overnight to be disinfected. Then, all samples were covered with laminin (1:100 dilution with PBS) for 4 h in an incubator. Next, the samples were put into 48-well plates and cultured with PC12 cell suspension for 24 h in RPMI1640 medium which containing 1% penicillin–streptomycin and 10% FBS. After 24 h, the samples were transferred to RPMI1640 medium composed of 1% HS, 1% penicillin–streptomycin and NGF (50 µg mL^−1^) for up to 7 days.

When testing the biological toxicity, the prepared substrates with cells were first placed in a 48-well plate before incubated in medium consisting of NGF for 24 h. Next, 450 μl of new medium and 50 μl of MTT solution were added to each well and put into an incubator for another 4 h. After the medium was cleared, 500 μl of DMSO was added in each well before further OD value measurement. As to the test of cell adhesion properties, the difference existed that after cultured with cells for 24 h, the substrates needed to be transferred to another new 48-well plate with medium consisting of NGF for the following operations.

In order to take immunofluorescence images of the cells, 4% paraformaldehyde was used to fix these substrates with cells for 30 min at room temperature after being cultured 3, 5, and 7 days in the medium containing NGF. Then, the solution of PBS and 0.1% Triton-X100 was utilized to permeabilize cytomembrane and fix protein for 1 h at room temperature. Next, the immobilized samples were stained with a mouse anti-βIII-tubulin primary antibody (1:800 dilution with PBT-1) overnight at 4 °C. PBT-1 consisted of 1% (v/v) Triton-X100, 1% BSA (Biofroxx, Germany), 5% (v/v) Donkey serum (Solarbio), 0.02% sodium azide (Sigma, USA) and 94% (v/v) PBS. Then, they were washed 3 times with the solution of PBS and 0.1% Triton-X100 and counterstained with a mixture of secondary antibodies (1:400 dilution with PBT-2) and DAPI (1:800 dilution with PBT-2) for 1 h at room temperature to label the target protein. PBT-2 consisted of 1% (v/v) Triton-X100 and 1% BSA. Ultimately, a DAKO fluorescence mounting medium was used to protect the samples from quenching before they were covered with coverslips.

Before SEM characterization, the substrates with cells were first washed 3 times in PBS and immobilized in 2.5 wt% glutaraldehyde solution at 4 °C overnight. Then, they were washed with deionized water 3 times to wash off the residual glutaraldehyde solution. Next, they were dehydrated through ethanol with a concentration gradient of 30%, 50%, 70% and 90% for 10 min respectively. Finally, they were dehydrated three times with 100% alcohol to make sure that the samples could be completely dehydrated before taking SEM images.

### Characterization

The inverse opal substrates were stretched by a vernier caliper (Masterproof, Germany). A digital multimeter (KEITHLEY, USA) was used to acquire the real-time resistance changes of the samples. As to MTT analysis, the absorbance of each well at 490 nm was measured by using a microplate reader (Synergy HT, BioTek, USA). SEM images were taken by a scanning electron microscope (S-3000 N, Hitachi, Japan). Confocal microscopy images were obtained by a Zeiss LSM700 laser-scanning microscope (Zeiss, Heidenheim, Germany).

## Conclusions

In summary, we have demonstrated a conductive inverse opal film with anisotropic elliptical porous patterns that can promote the growth of neurites and induce their directional arrangement. The film is derived from a tensile polymer inverse opal structure and covered with PEDOT:PSS-doped PAAm composite hydrogels. Its great conductivity and biocompatibility can make neurites extend well and fully connected. Meanwhile, the anisotropic properties of the stretched films provide possibilities to induce the directional growth of nerve cells connected with the stretching orientation. We can conveniently induce nerve cells to grow in different orientations by simply changing the stretching direction. Apart from on the 0°-stretched films, the cells seeded on the films stretched at 15°, 30° and 45° grow in two crossing directions due to the special surface topography of the films, forming a neural network for the connection of nerve cells. In addition, the composite hydrogel film has an advantage over nerve cells differentiation even without external electrical stimulation. Therefore, these characteristics of this conductive inverse opal film have broad application prospects in tissue engineering.

## Supplementary Information

**Additional file 1**: **Figure S1.** SEM images of (a) the silica colloidal crystal template, (b) the PS hybrid colloidal crystal template, (c) the PS inverse opal film. Scale bars are 500 nm. **Figure S2.** Different stretching degrees. (a) 3-times, (b) 6-times, (c) 9-times, (d) 12-times stretched PS inverse opal films. Scale bars are 1 μm. **Figure S3.** (a) MTT assays and (b) adhesion properties of PC12 cells cultured on ordinary glass slides, PS substrates stretched at 0°, 15°, 30°, 45° for 1 day, 2 days, and 3 days, respectively. Error bars represent SD. **Figure S4.** (a) Immunofluorescence image, (b) SEM image, (c) angle distribution of neurites of PC12 cells cultured on ordinary glass slides. Scale bars are 50 μm. **Figure S5.** Orientation angle frequency distribution of PC12 cells cultured on PS inverse opal films stretched at different angles. *θ* or *θ*’ means the angle between the direction of neurite (the red dotted line) and the stretching orientation (the black solid line), respectively. **Figure S6.** Raman spectrum of PEDOT:PSS-doped PAAm hydrogels. **Figure S7.** (a) MTT assays and (b) adhesion properties of PC12 cells cultured on ordinary glass slides, PS inverse opal films, composite films for 1 day, 2 days, and 3 days, respectively. Error bars represent SD. **Figure S8.** (a) Differentiation rates of PC12 cells cultured on ordinary glass slides, PS inverse opal films and composite films on the 7th day. (b) Orientation angle frequency distribution of PC12 cells on PS inverse opal films and composite films.

## Data Availability

All data generated or analyzed during this study are included in this manuscript and its additional file.
