# Supplementary material for: Tailoring conductive inverse opal films with anisotropic elliptical porous patterns for nerve cell orientation
Source: J Nanobiotechnology. 2022 Mar 9;20:117. doi: 10.1186/s12951-022-01340-w (PMC8905848; doi:10.1186/s12951-022-01340-w)
Supplement: Supplementary file 1 — Additional file 1: Figure S1. SEM images of (a) the silica colloidal crystal template, (b) the PS hybrid colloidal crystal template, (c) the PS inverse opal film. Scale bars are 500 nm. Figure S2. Different stretching degrees. (a) 3-times, (b) 6-times, (c) 9-times, (d) 12-times stretched PS inverse opal films. Scale bars are 1 μm. Figure S3. (a) MTT assays and (b) adhesion properties of PC12 cells cultured on ordinary glass slides, PS substrates stretched at 0°, 15°, 30°, 45° for 1 day, 2 days, and 3 days, respectively. Error bars represent SD. Figure S4. (a) Immunofluorescence image, (b) SEM image, (c) angle distribution of neurites of PC12 cells cultured on ordinary glass slides. Scale bars are 50 μm. Figure S5. Orientation angle frequency distribution of PC12 cells cultured on PS inverse opal films stretched at different angles. θ or θ’ means the angle between the direction of neurite (the red dotted line) and the stretching orientation (the black solid line), respectively. Figure S6. Raman spectrum of PEDOT:PSS-doped PAAm hydrogels. Figure S7. (a) MTT assays and (b) adhesion properties of PC12 cells cultured on ordinary glass slides, PS inverse opal films, composite films for 1 day, 2 days, and 3 days, respectively. Error bars represent SD. Figure S8. (a) Differentiation rates of PC12 cells cultured on ordinary glass slides, PS inverse opal films and composite films on the 7th day. (b) Orientation angle frequency distribution of PC12 cells on PS inverse opal films and composite films. [file 12951_2022_1340_MOESM1_ESM.docx]

Additional file 1 for

**Tailoring conductive inverse opal films with anisotropic elliptical porous patterns for nerve cell orientation**

Zeyou Zhang^1,2^, Yu Wang^1^, Zhuoyue Chen^2^, Dongyu Xu^2^, Dagan Zhang^1,2,*^, Fengyuan Wang^2,3,*^, Yuanjin Zhao^1,2,*^

^1^ Department of Clinical Laboratory, Institute of Translational Medicine, The Affiliated Drum Tower Hospital of Nanjing University Medical School, Nanjing 210008, China

^2^ State Key Laboratory of Bioelectronics, School of Biological Science and Medical Engineering, Southeast University, Nanjing 210096, China

^3^ Department of Dermatology, Zhongda Hospital, Southeast University, Nanjing 210009, China


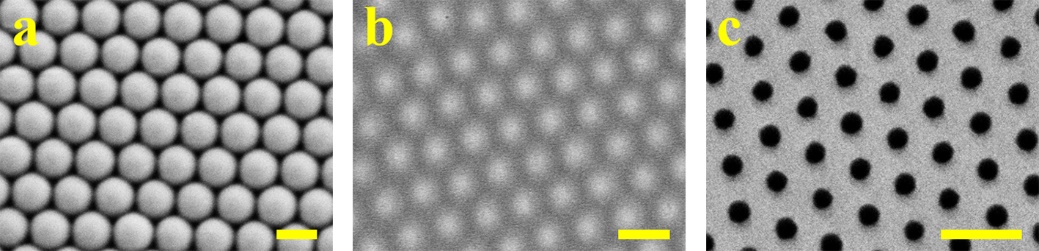


**Figure S1.** SEM images of (a) the silica colloidal crystal template, (b) the PS hybrid colloidal crystal template, (c) the PS inverse opal film. Scale bars are 500 nm.


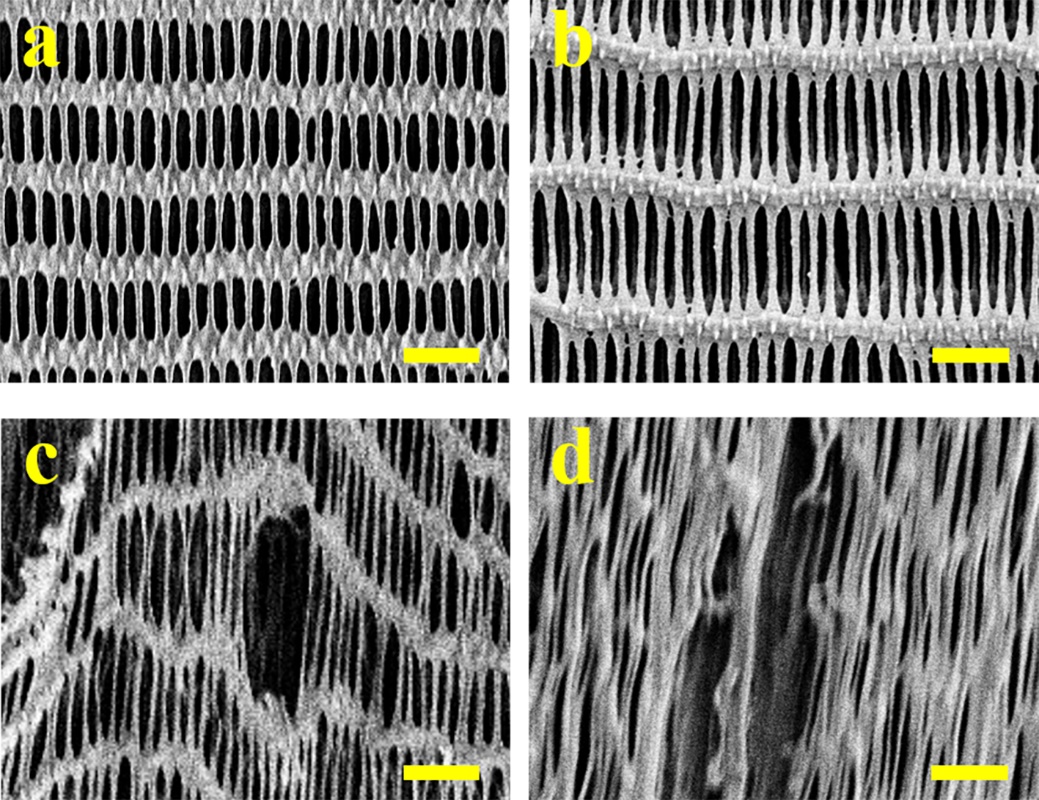


**Figure S2.** Different stretching degrees. (a) 3-times, (b) 6-times, (c) 9-times, (d) 12-times stretched PS inverse opal films. Scale bars are 1 μm.


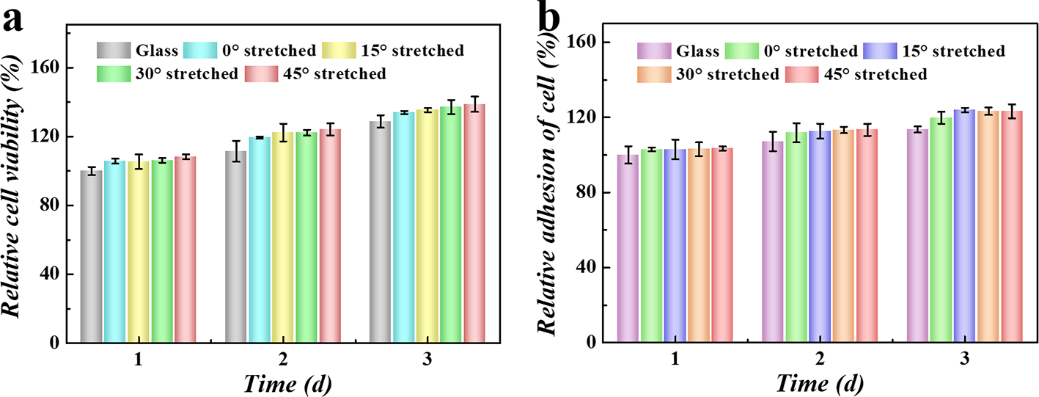


**Figure S3.** (a) MTT assays and (b) adhesion properties of PC12 cells cultured on ordinary glass slides, PS substrates stretched at 0°, 15°, 30°, 45° for 1 day, 2 days, and 3 days, respectively. Error bars represent SD.


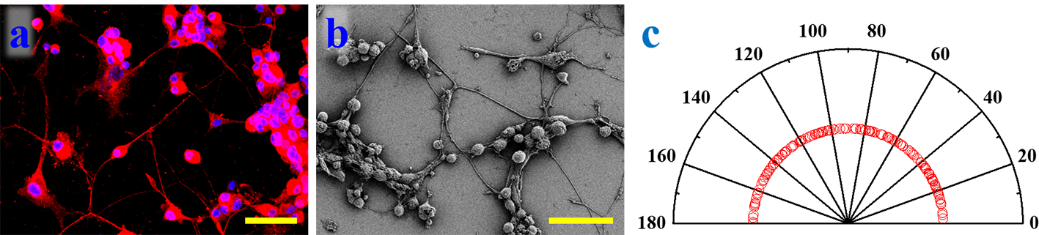


**Figure S4.** (a) Immunofluorescence image, (b) SEM image, (c) angle distribution of neurites of PC12 cells cultured on ordinary glass slides. Scale bars are 50 μm.


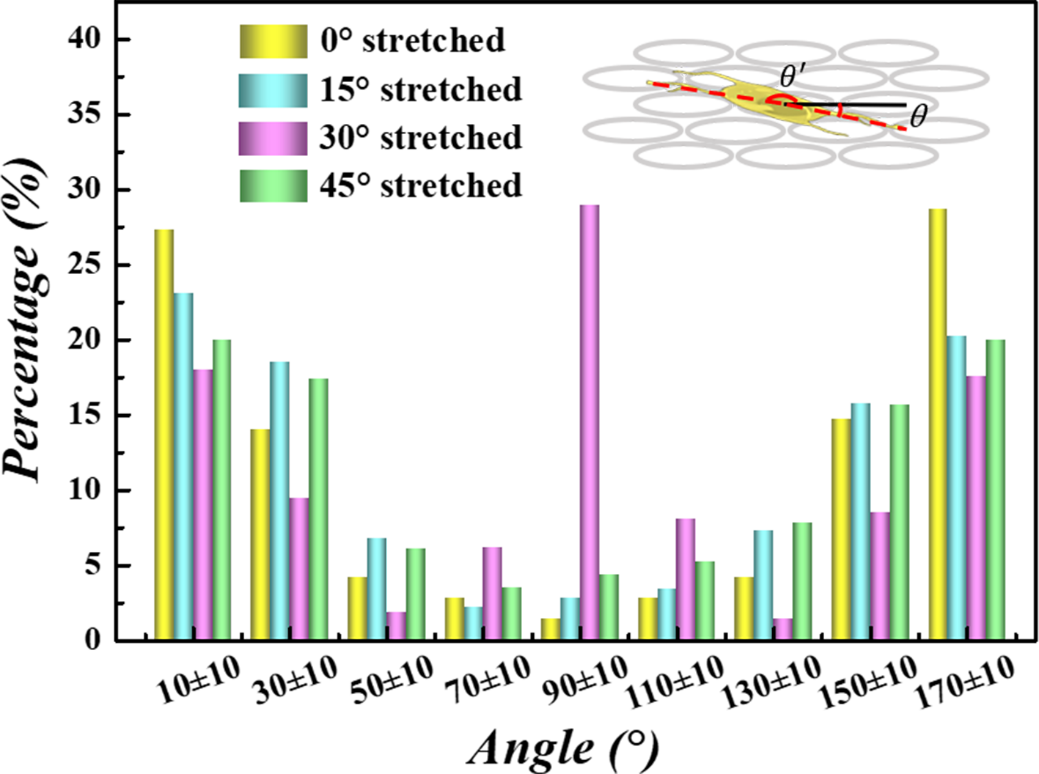


**Figure S5.** Orientation angle frequency distribution of PC12 cells cultured on PS inverse opal films stretched at different angles. *θ* or *θ’* means the angle between the direction of neurite (the red dotted line) and the stretching orientation (the black solid line), respectively.


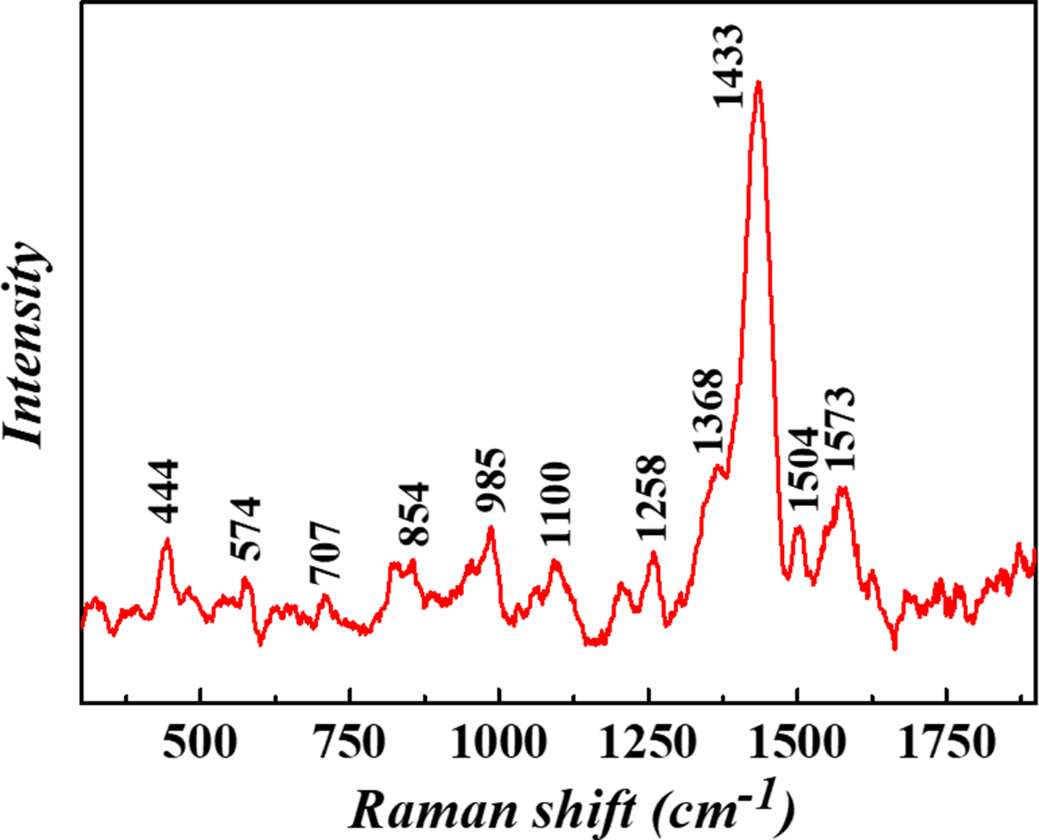


**Figure S6.** Raman spectrum of PEDOT:PSS-doped PAAm hydrogels.


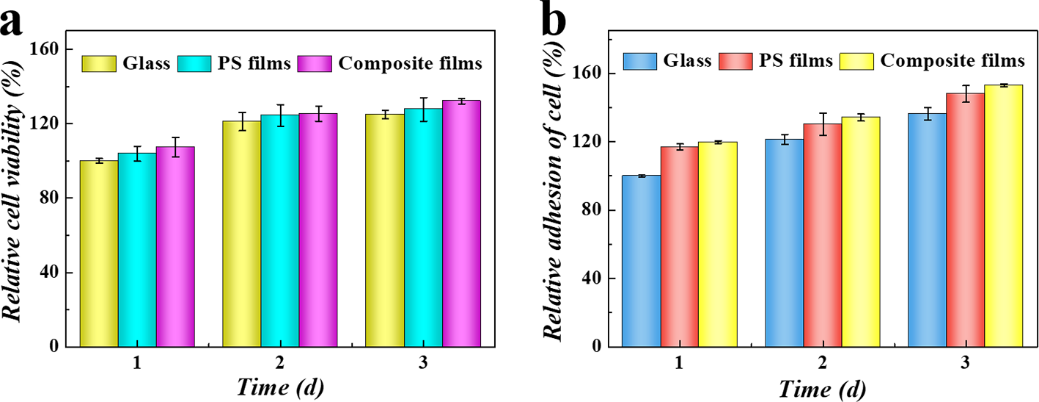


**Figure S7.** (a) MTT assays and (b) adhesion properties of PC12 cells cultured on ordinary glass slides, PS inverse opal films, composite films for 1 day, 2 days, and 3 days, respectively. Error bars represent SD.


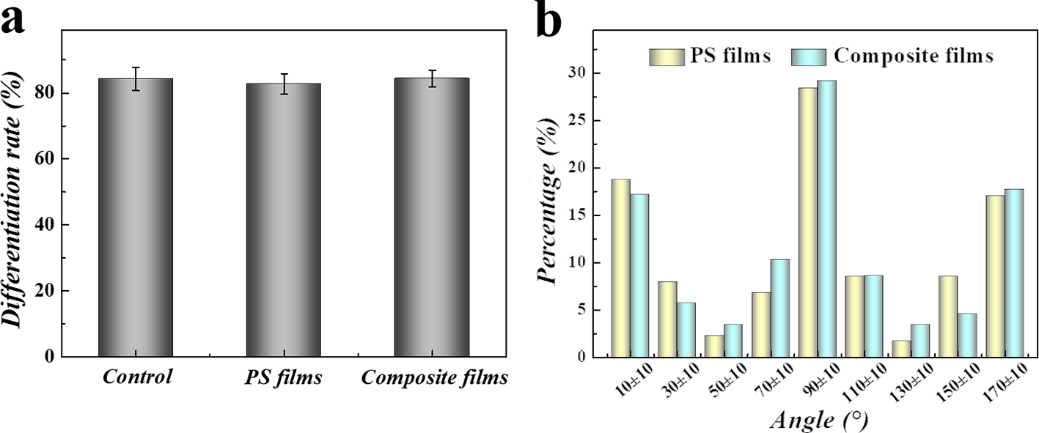


**Figure S8.** (a) Differentiation rates of PC12 cells cultured on ordinary glass slides, PS inverse opal films and composite films on the 7th day. (b) Orientation angle frequency distribution of PC12 cells on PS inverse opal films and composite films.
